# *rac*-(3-Hydr­oxy-2-phenyl­propionato-κ*O*)triphenyl­tin(IV)

**DOI:** 10.1107/S160053680803242X

**Published:** 2008-10-15

**Authors:** Mostafa M. Amini, Taraneh Hajiashrafi, Ali Nemati Kharat, Seik Weng Ng

**Affiliations:** a Department of Chemistry, Shahid Beheshti University, Tehran, Iran; b School of Chemistry, College of Science, Tehran University, Tehran, Iran; c Department of Chemistry, University of Malaya, 50603 Kuala Lumpur, Malaysia

## Abstract

The Sn^IV^ atom in the monomeric title compound, [Sn(C_6_H_5_)_3_(C_9_H_9_O_3_)] exists in a distorted SnC_3_O tetra­hedral geometry. In the crystal structure, inversion dimers arise from pairs of O—H⋯O hydrogen bonds.

## Related literature

For reviews of organotin carboxyl­ates, see: Tiekink (1991 ▶, 1994 ▶).
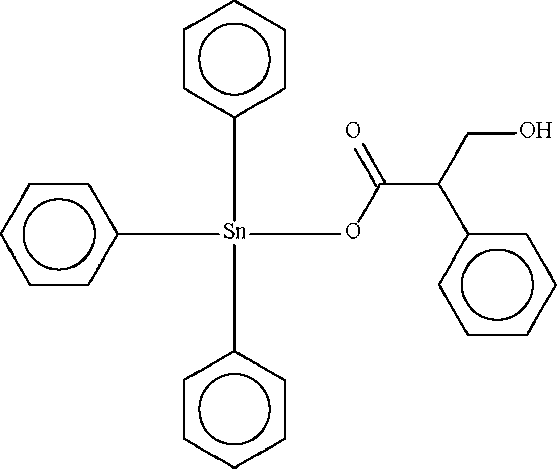


## Experimental

### 

#### Crystal data

[Sn(C_6_H_5_)_3_(C_9_H_9_O_3_)]
*M* *_r_* = 515.15
Triclinic, 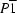

*a* = 9.3880 (2) Å
*b* = 9.4899 (2) Å
*c* = 14.3399 (2) Å
α = 90.087 (1)°
β = 103.664 (1)°
γ = 112.758 (1)°
*V* = 1138.58 (4) Å^3^
*Z* = 2
Mo *K*α radiation
μ = 1.15 mm^−1^
*T* = 112 (2) K
0.39 × 0.32 × 0.31 mm

#### Data collection

Bruker APEXII diffractometer
Absorption correction: multi-scan (*SADABS*; Sheldrick, 1996 ▶) *T*_min_ = 0.663, *T*_max_ = 0.717
33531 measured reflections
6612 independent reflections
6339 reflections with *I* > 2σ(*I*)
*R*_int_ = 0.020

#### Refinement

*R*[*F* ^2^ > 2σ(*F* ^2^)] = 0.019
*wR*(*F* ^2^) = 0.054
*S* = 1.05
6612 reflections
281 parameters
H-atom parameters constrained
Δρ_max_ = 0.64 e Å^−3^
Δρ_min_ = −0.35 e Å^−3^

### 

Data collection: *APEX2* (Bruker, 2004 ▶); cell refinement: *SAINT* (Bruker, 2004 ▶); data reduction: *SAINT*; program(s) used to solve structure: *SHELXS97* (Sheldrick, 2008 ▶); program(s) used to refine structure: *SHELXL97* (Sheldrick, 2008 ▶); molecular graphics: *X-SEED* (Barbour, 2001 ▶); software used to prepare material for publication: *publCIF* (Westrip, 2008 ▶).

## Supplementary Material

Crystal structure: contains datablocks global, I. DOI: 10.1107/S160053680803242X/hb2813sup1.cif

Structure factors: contains datablocks I. DOI: 10.1107/S160053680803242X/hb2813Isup2.hkl

Additional supplementary materials:  crystallographic information; 3D view; checkCIF report

## Figures and Tables

**Table 1:** Selected bond lengths (Å)

Sn1—O1	2.082 (1)
Sn1—C1	2.123 (1)
Sn1—C7	2.122 (1)
Sn1—C13	2.128 (1)

**Table 2:** Hydrogen-bond geometry (Å, °)

*D*—H⋯*A*	*D*—H	H⋯*A*	*D*⋯*A*	*D*—H⋯*A*
O3—H3⋯O2^i^	0.84	1.98	2.819 (2)	175

Symmetry code: (i) 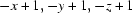.

**Fig. 1.**
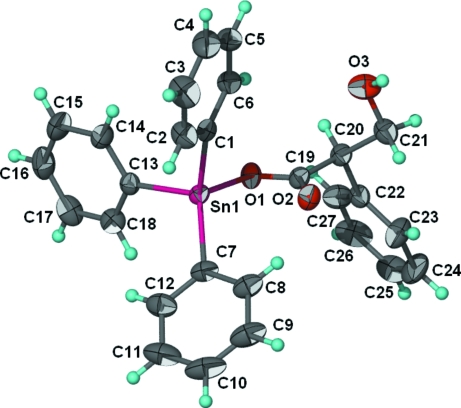
The molecular structure of (I) showing displacement ellipsoids at the 70% probability level; hydrogen atoms are shown as spheres of arbitrary radius.

## supplementary crystallographic information

### Comment

The monomeric structure found for triphenyltin 2-hydroxy-3-phenylpropionate (Scheme I, Fig. 1) conforms to expectations based on the presence of bulky substituents (Tiekink, 1991, 1994). In the arbitrarily chosen asymmetric unit, C20 has R configuration, but crystal symmetry generates a racemic mixture. Selected geometrical data are given in Tables 1 and 2.

### Experimental

Triphenyltin hydroxide (1.0 g, 2.7 mmol) and *d*,*l*-tropic acid (0.45 g, 2.7 mmol) were heated in toluene (100 ml) in a Dean–Stark water-separator until all the water had been removed. The solvent was removed under reduced pressure to leave a white solid. The solid was recrystallized from mixture of chloroform, hexane and toluene (2:1:1 *v*/*v*) to give colorless blocks of (I), m.p. 385–386 K.

IR (KBr, cm^-1^): 3449 (OH), 1627 (CO, *asym*), 1355 (CO, *sym*), 576, 601 (Sn—C). ^1^H NMR (CDCl_3_): 3.72–4.08 (m, 3H), 7.28–7.78 (20H, C_6_H_5_) p.p.m. ^13^C NMR (CDCl_3_): 53.90 (CH), 65.08 (CH_2_) 127.5–137.7 (C_6_H_5_) p.p.m. ^119^Sn NMR (CDCl_3_): -83.1 p.p.m. Mass spectrum (*m*/*e*): 515 (M–1) [Ph_3_SnO_2_CCH(CH_2_O)Ph]^+^, 439 (M–Ph) [Ph_3_SnO_2_CCH(CH_2_OH)]^+^.

### Refinement

The hydrogen atoms were placed in calculated positions (C—H = 0.95–1.00 Å, O—H = 0.84 Å) and refined as riding with U_iso_(H) = 1.2U_eq_(carrier).

### Crystal data

**Table:**

[Sn(C_6_H_5_)_3_(C_9_H_9_O_3_)]	*Z* = 2
*M**_r_* = 515.15	*F*(000) = 520
Triclinic, *P*1	*D*_x_ = 1.503 Mg m^−^^3^
Hall symbol: -P 1	Mo *K*α radiation, λ = 0.71073 Å
*a* = 9.3880 (2) Å	Cell parameters from 9425 reflections
*b* = 9.4899 (2) Å	θ = 2.3–32.6°
*c* = 14.3399 (2) Å	µ = 1.15 mm^−^^1^
α = 90.087 (1)°	*T* = 112 K
β = 103.664 (1)°	Block, colourless
γ = 112.758 (1)°	0.39 × 0.32 × 0.31 mm
*V* = 1138.58 (4) Å^3^	

### Data collection

**Table:**

Bruker APEXII diffractometer	6612 independent reflections
Radiation source: medium-focus sealed tube	6339 reflections with *I* > 2σ(*I*)
graphite	*R*_int_ = 0.020
φ and ω scans	θ_max_ = 30.0°, θ_min_ = 1.5°
Absorption correction: multi-scan (SADABS; Sheldrick, 1996)	*h* = −13→13
*T*_min_ = 0.663, *T*_max_ = 0.717	*k* = −13→13
33531 measured reflections	*l* = −19→20

### Refinement

**Table:**

Refinement on *F*^2^	Primary atom site location: structure-invariant direct methods
Least-squares matrix: full	Secondary atom site location: difference Fourier map
*R*[*F*^2^ > 2σ(*F*^2^)] = 0.019	Hydrogen site location: inferred from neighbouring sites
*wR*(*F*^2^) = 0.054	H-atom parameters constrained
*S* = 1.05	*w* = 1/[σ^2^(*F*_o_^2^) + (0.0304*P*)^2^ + 0.5255*P*] where *P* = (*F*_o_^2^ + 2*F*_c_^2^)/3
6612 reflections	(Δ/σ)_max_ = 0.001
281 parameters	Δρ_max_ = 0.64 e Å^−^^3^
0 restraints	Δρ_min_ = −0.35 e Å^−^^3^

### Fractional atomic coordinates and isotropic or equivalent isotropic displacement parameters (Å^2^)

**Table:**

	*x*	*y*	*z*	*U*_iso_*/*U*_eq_	
Sn1	0.588742 (10)	0.636637 (10)	0.826066 (6)	0.02043 (3)	
O1	0.74512 (13)	0.76508 (12)	0.74695 (7)	0.0268 (2)	
O2	0.52531 (13)	0.67054 (13)	0.62949 (8)	0.0303 (2)	
O3	0.71606 (16)	0.55344 (13)	0.51412 (10)	0.0394 (3)	
H3	0.6449	0.4826	0.4734	0.059*	
C1	0.54423 (16)	0.40565 (15)	0.78448 (9)	0.0222 (2)	
C2	0.4203 (2)	0.28743 (18)	0.81068 (12)	0.0330 (3)	
H2	0.3515	0.3111	0.8411	0.040*	
C3	0.3973 (3)	0.1349 (2)	0.79226 (15)	0.0441 (4)	
H3A	0.3129	0.0549	0.8102	0.053*	
C4	0.4965 (2)	0.09935 (19)	0.74803 (13)	0.0400 (4)	
H4	0.4812	−0.0048	0.7365	0.048*	
C5	0.6186 (2)	0.21545 (19)	0.72034 (12)	0.0334 (3)	
H5	0.6856	0.1908	0.6888	0.040*	
C6	0.64278 (18)	0.36822 (17)	0.73878 (11)	0.0274 (3)	
H6	0.7269	0.4476	0.7201	0.033*	
C7	0.38486 (17)	0.68487 (16)	0.82227 (12)	0.0275 (3)	
C8	0.25851 (18)	0.64580 (18)	0.73831 (13)	0.0333 (3)	
H8	0.2686	0.6072	0.6802	0.040*	
C9	0.1186 (2)	0.6635 (2)	0.74001 (16)	0.0425 (4)	
H9	0.0335	0.6372	0.6831	0.051*	
C10	0.1040 (2)	0.7191 (2)	0.82417 (19)	0.0480 (5)	
H10	0.0082	0.7305	0.8252	0.058*	
C11	0.2273 (2)	0.7587 (2)	0.90783 (18)	0.0458 (4)	
H11	0.2160	0.7978	0.9655	0.055*	
C12	0.3687 (2)	0.74112 (19)	0.90717 (14)	0.0358 (3)	
H12	0.4531	0.7675	0.9644	0.043*	
C13	0.76077 (16)	0.71155 (16)	0.96187 (10)	0.0231 (2)	
C14	0.8361 (2)	0.61586 (19)	1.00105 (11)	0.0323 (3)	
H14	0.8035	0.5161	0.9696	0.039*	
C15	0.9590 (2)	0.6657 (2)	1.08601 (13)	0.0427 (4)	
H15	1.0100	0.6001	1.1120	0.051*	
C16	1.0063 (2)	0.8105 (2)	1.13220 (12)	0.0407 (4)	
H16	1.0911	0.8451	1.1894	0.049*	
C17	0.9304 (2)	0.9052 (2)	1.09543 (12)	0.0366 (3)	
H17	0.9617	1.0038	1.1282	0.044*	
C18	0.80848 (19)	0.85653 (17)	1.01073 (11)	0.0296 (3)	
H18	0.7571	0.9223	0.9858	0.036*	
C19	0.67104 (17)	0.73973 (16)	0.65568 (10)	0.0242 (2)	
C20	0.77747 (17)	0.80071 (16)	0.58653 (10)	0.0240 (2)	
H20	0.8833	0.7969	0.6167	0.029*	
C21	0.7052 (2)	0.69608 (18)	0.49250 (11)	0.0310 (3)	
H21A	0.5922	0.6808	0.4664	0.037*	
H21B	0.7649	0.7415	0.4441	0.037*	
C22	0.80597 (18)	0.96785 (16)	0.57297 (10)	0.0248 (3)	
C23	0.6983 (2)	1.0097 (2)	0.50719 (13)	0.0394 (4)	
H23	0.6010	0.9326	0.4703	0.047*	
C24	0.7320 (3)	1.1637 (2)	0.49495 (15)	0.0481 (5)	
H24	0.6583	1.1908	0.4490	0.058*	
C25	0.8706 (3)	1.2768 (2)	0.54866 (16)	0.0439 (4)	
H25	0.8932	1.3818	0.5399	0.053*	
C26	0.9769 (2)	1.2367 (2)	0.61539 (17)	0.0444 (4)	
H26	1.0719	1.3146	0.6538	0.053*	
C27	0.9458 (2)	1.08338 (19)	0.62678 (13)	0.0348 (3)	
H27	1.0212	1.0571	0.6719	0.042*	

### Atomic displacement parameters (Å^2^)

**Table:**

	*U*^11^	*U*^22^	*U*^33^	*U*^12^	*U*^13^	*U*^23^
Sn1	0.01937 (5)	0.01980 (5)	0.02268 (5)	0.00779 (3)	0.00640 (3)	0.00547 (3)
O1	0.0292 (5)	0.0248 (5)	0.0234 (4)	0.0066 (4)	0.0084 (4)	0.0063 (4)
O2	0.0288 (5)	0.0332 (5)	0.0280 (5)	0.0116 (4)	0.0067 (4)	0.0078 (4)
O3	0.0394 (6)	0.0223 (5)	0.0536 (7)	0.0103 (5)	0.0104 (6)	−0.0018 (5)
C1	0.0223 (6)	0.0217 (6)	0.0209 (5)	0.0075 (5)	0.0044 (4)	0.0048 (4)
C2	0.0350 (8)	0.0257 (7)	0.0369 (8)	0.0060 (6)	0.0173 (6)	0.0051 (6)
C3	0.0518 (11)	0.0232 (7)	0.0515 (10)	0.0028 (7)	0.0239 (9)	0.0067 (7)
C4	0.0555 (11)	0.0224 (7)	0.0398 (8)	0.0140 (7)	0.0108 (8)	0.0027 (6)
C5	0.0403 (8)	0.0319 (7)	0.0325 (7)	0.0193 (7)	0.0089 (6)	0.0017 (6)
C6	0.0268 (6)	0.0253 (6)	0.0306 (7)	0.0099 (5)	0.0093 (5)	0.0052 (5)
C7	0.0222 (6)	0.0217 (6)	0.0409 (8)	0.0092 (5)	0.0115 (6)	0.0083 (5)
C8	0.0246 (7)	0.0277 (7)	0.0467 (9)	0.0106 (6)	0.0073 (6)	0.0088 (6)
C9	0.0257 (7)	0.0337 (8)	0.0679 (12)	0.0139 (6)	0.0081 (8)	0.0113 (8)
C10	0.0301 (8)	0.0326 (8)	0.0894 (16)	0.0169 (7)	0.0221 (9)	0.0141 (9)
C11	0.0437 (10)	0.0322 (8)	0.0731 (13)	0.0175 (7)	0.0318 (10)	0.0050 (8)
C12	0.0335 (8)	0.0299 (7)	0.0485 (9)	0.0126 (6)	0.0186 (7)	0.0053 (7)
C13	0.0240 (6)	0.0240 (6)	0.0216 (5)	0.0086 (5)	0.0082 (5)	0.0050 (5)
C14	0.0395 (8)	0.0328 (7)	0.0267 (7)	0.0191 (7)	0.0040 (6)	0.0042 (6)
C15	0.0461 (10)	0.0534 (11)	0.0311 (8)	0.0281 (9)	0.0000 (7)	0.0070 (7)
C16	0.0326 (8)	0.0551 (11)	0.0251 (7)	0.0110 (8)	0.0016 (6)	0.0002 (7)
C17	0.0330 (8)	0.0321 (8)	0.0335 (8)	0.0014 (6)	0.0084 (6)	−0.0048 (6)
C18	0.0292 (7)	0.0246 (6)	0.0326 (7)	0.0075 (5)	0.0089 (6)	0.0032 (5)
C19	0.0301 (6)	0.0199 (6)	0.0240 (6)	0.0107 (5)	0.0083 (5)	0.0067 (5)
C20	0.0238 (6)	0.0228 (6)	0.0255 (6)	0.0083 (5)	0.0080 (5)	0.0061 (5)
C21	0.0367 (8)	0.0260 (7)	0.0300 (7)	0.0096 (6)	0.0133 (6)	0.0034 (5)
C22	0.0308 (7)	0.0214 (6)	0.0237 (6)	0.0090 (5)	0.0122 (5)	0.0063 (5)
C23	0.0438 (9)	0.0311 (8)	0.0345 (8)	0.0124 (7)	−0.0016 (7)	0.0065 (6)
C24	0.0682 (13)	0.0386 (9)	0.0429 (10)	0.0293 (9)	0.0105 (9)	0.0182 (8)
C25	0.0600 (11)	0.0237 (7)	0.0595 (11)	0.0159 (8)	0.0375 (10)	0.0160 (7)
C26	0.0344 (8)	0.0268 (8)	0.0681 (13)	0.0036 (6)	0.0210 (8)	−0.0049 (8)
C27	0.0306 (7)	0.0296 (7)	0.0431 (9)	0.0118 (6)	0.0075 (6)	−0.0005 (6)

### Geometric parameters (Å, °)

**Table:**

Sn1—O1	2.082 (1)	C12—H12	0.9500
Sn1—C1	2.123 (1)	C13—C18	1.399 (2)
Sn1—C7	2.122 (1)	C13—C14	1.397 (2)
Sn1—C13	2.128 (1)	C14—C15	1.397 (2)
O1—C19	1.3016 (17)	C14—H14	0.9500
O2—C19	1.2269 (18)	C15—C16	1.383 (3)
O3—C21	1.4254 (19)	C15—H15	0.9500
O3—H3	0.8400	C16—C17	1.383 (3)
C1—C6	1.397 (2)	C16—H16	0.9500
C1—C2	1.3970 (19)	C17—C18	1.390 (2)
C2—C3	1.394 (2)	C17—H17	0.9500
C2—H2	0.9500	C18—H18	0.9500
C3—C4	1.380 (3)	C19—C20	1.5294 (19)
C3—H3A	0.9500	C20—C22	1.5240 (19)
C4—C5	1.387 (3)	C20—C21	1.519 (2)
C4—H4	0.9500	C20—H20	1.0000
C5—C6	1.392 (2)	C21—H21A	0.9900
C5—H5	0.9500	C21—H21B	0.9900
C6—H6	0.9500	C22—C23	1.389 (2)
C7—C12	1.392 (2)	C22—C27	1.388 (2)
C7—C8	1.406 (2)	C23—C24	1.392 (2)
C8—C9	1.393 (2)	C23—H23	0.9500
C8—H8	0.9500	C24—C25	1.372 (3)
C9—C10	1.373 (3)	C24—H24	0.9500
C9—H9	0.9500	C25—C26	1.378 (3)
C10—C11	1.388 (3)	C25—H25	0.9500
C10—H10	0.9500	C26—C27	1.387 (2)
C11—C12	1.402 (2)	C26—H26	0.9500
C11—H11	0.9500	C27—H27	0.9500
			
O1—Sn1—C1	104.08 (5)	C15—C14—H14	119.7
O1—Sn1—C7	117.51 (5)	C13—C14—H14	119.7
O1—Sn1—C13	94.95 (5)	C16—C15—C14	119.92 (17)
C1—Sn1—C7	116.29 (5)	C16—C15—H15	120.0
C1—Sn1—C13	109.17 (5)	C14—C15—H15	120.0
C7—Sn1—C13	112.54 (6)	C15—C16—C17	120.18 (16)
C19—O1—Sn1	109.50 (9)	C15—C16—H16	119.9
C21—O3—H3	109.5	C17—C16—H16	119.9
C6—C1—C2	118.99 (13)	C18—C17—C16	120.14 (16)
C6—C1—Sn1	122.04 (10)	C18—C17—H17	119.9
C2—C1—Sn1	118.77 (11)	C16—C17—H17	119.9
C3—C2—C1	120.14 (15)	C17—C18—C13	120.60 (15)
C3—C2—H2	119.9	C17—C18—H18	119.7
C1—C2—H2	119.9	C13—C18—H18	119.7
C4—C3—C2	120.38 (16)	O2—C19—O1	120.51 (13)
C4—C3—H3A	119.8	O2—C19—C20	123.92 (13)
C2—C3—H3A	119.8	O1—C19—C20	115.57 (12)
C3—C4—C5	120.09 (15)	C22—C20—C21	113.37 (12)
C3—C4—H4	120.0	C22—C20—C19	110.50 (11)
C5—C4—H4	120.0	C21—C20—C19	109.60 (12)
C6—C5—C4	119.92 (15)	C22—C20—H20	107.7
C6—C5—H5	120.0	C21—C20—H20	107.7
C4—C5—H5	120.0	C19—C20—H20	107.7
C5—C6—C1	120.48 (14)	O3—C21—C20	106.66 (13)
C5—C6—H6	119.8	O3—C21—H21A	110.4
C1—C6—H6	119.8	C20—C21—H21A	110.4
C12—C7—C8	119.39 (15)	O3—C21—H21B	110.4
C12—C7—Sn1	119.13 (12)	C20—C21—H21B	110.4
C8—C7—Sn1	121.13 (12)	H21A—C21—H21B	108.6
C9—C8—C7	120.32 (17)	C23—C22—C27	118.32 (14)
C9—C8—H8	119.8	C23—C22—C20	122.50 (14)
C7—C8—H8	119.8	C27—C22—C20	119.17 (14)
C10—C9—C8	119.76 (18)	C22—C23—C24	120.45 (17)
C10—C9—H9	120.1	C22—C23—H23	119.8
C8—C9—H9	120.1	C24—C23—H23	119.8
C11—C10—C9	120.90 (17)	C25—C24—C23	120.65 (18)
C11—C10—H10	119.5	C25—C24—H24	119.7
C9—C10—H10	119.5	C23—C24—H24	119.7
C10—C11—C12	119.91 (19)	C24—C25—C26	119.37 (16)
C10—C11—H11	120.0	C24—C25—H25	120.3
C12—C11—H11	120.0	C26—C25—H25	120.3
C7—C12—C11	119.71 (18)	C25—C26—C27	120.40 (18)
C7—C12—H12	120.1	C25—C26—H26	119.8
C11—C12—H12	120.1	C27—C26—H26	119.8
C18—C13—C14	118.58 (14)	C26—C27—C22	120.79 (17)
C18—C13—Sn1	122.64 (11)	C26—C27—H27	119.6
C14—C13—Sn1	118.67 (11)	C22—C27—H27	119.6
C15—C14—C13	120.55 (16)		
			
C1—Sn1—O1—C19	67.61 (10)	C7—Sn1—C13—C18	−45.80 (13)
C7—Sn1—O1—C19	−62.58 (11)	O1—Sn1—C13—C14	−99.31 (12)
C13—Sn1—O1—C19	178.80 (10)	C1—Sn1—C13—C14	7.46 (13)
O1—Sn1—C1—C6	17.13 (12)	C7—Sn1—C13—C14	138.15 (12)
C7—Sn1—C1—C6	148.04 (11)	C18—C13—C14—C15	−1.5 (2)
C13—Sn1—C1—C6	−83.32 (12)	Sn1—C13—C14—C15	174.73 (14)
O1—Sn1—C1—C2	−168.17 (11)	C13—C14—C15—C16	0.3 (3)
C7—Sn1—C1—C2	−37.26 (13)	C14—C15—C16—C17	1.1 (3)
C13—Sn1—C1—C2	91.39 (12)	C15—C16—C17—C18	−1.4 (3)
C6—C1—C2—C3	0.7 (2)	C16—C17—C18—C13	0.2 (2)
Sn1—C1—C2—C3	−174.13 (14)	C14—C13—C18—C17	1.2 (2)
C1—C2—C3—C4	0.0 (3)	Sn1—C13—C18—C17	−174.81 (12)
C2—C3—C4—C5	−0.9 (3)	Sn1—O1—C19—O2	11.24 (16)
C3—C4—C5—C6	1.1 (3)	Sn1—O1—C19—C20	−168.49 (9)
C4—C5—C6—C1	−0.4 (2)	O2—C19—C20—C22	93.95 (17)
C2—C1—C6—C5	−0.5 (2)	O1—C19—C20—C22	−86.33 (15)
Sn1—C1—C6—C5	174.18 (11)	O2—C19—C20—C21	−31.69 (19)
O1—Sn1—C7—C12	−116.37 (12)	O1—C19—C20—C21	148.03 (13)
C1—Sn1—C7—C12	119.36 (12)	C22—C20—C21—O3	168.43 (12)
C13—Sn1—C7—C12	−7.63 (14)	C19—C20—C21—O3	−67.59 (15)
O1—Sn1—C7—C8	70.48 (13)	C21—C20—C22—C23	39.8 (2)
C1—Sn1—C7—C8	−53.78 (13)	C19—C20—C22—C23	−83.70 (18)
C13—Sn1—C7—C8	179.23 (11)	C21—C20—C22—C27	−138.98 (15)
C12—C7—C8—C9	0.1 (2)	C19—C20—C22—C27	97.53 (16)
Sn1—C7—C8—C9	173.25 (12)	C27—C22—C23—C24	1.0 (3)
C7—C8—C9—C10	−0.1 (3)	C20—C22—C23—C24	−177.80 (17)
C8—C9—C10—C11	0.3 (3)	C22—C23—C24—C25	−1.1 (3)
C9—C10—C11—C12	−0.5 (3)	C23—C24—C25—C26	−0.1 (3)
C8—C7—C12—C11	−0.3 (2)	C24—C25—C26—C27	1.4 (3)
Sn1—C7—C12—C11	−173.55 (13)	C25—C26—C27—C22	−1.5 (3)
C10—C11—C12—C7	0.5 (3)	C23—C22—C27—C26	0.3 (3)
O1—Sn1—C13—C18	76.74 (12)	C20—C22—C27—C26	179.09 (15)
C1—Sn1—C13—C18	−176.49 (11)		

### Hydrogen-bond geometry (Å, °)

**Table:**

*D*—H···*A*	*D*—H	H···*A*	*D*···*A*	*D*—H···*A*
O3—H3···O2^i^	0.84	1.98	2.819 (2)	175

Symmetry codes: (i) −*x*+1, −*y*+1, −*z*+1.

## References

[bb1] Barbour, L. J. (2001). *J. Supramol. Chem.* **1**, 189–191.

[bb2] Bruker (2004). *APEX2* and *SAINT* Bruker AXS Inc., Madison, Wisconsin, USA.

[bb3] Sheldrick, G. M. (1996). *SADABS* University of Göttingen, Germany.

[bb4] Sheldrick, G. M. (2008). *Acta Cryst.* A**64**, 112–122. 10.1107/S0108767307043930 18156677

[bb5] Tiekink, E. R. T. (1991). *Appl. Organomet. Chem.* **5**, 1–23.

[bb6] Tiekink, E. R. T. (1994). *Trends Organomet. Chem.* **1**, 71–116.

[bb7] Westrip, S. P. (2008). *publCIF.* In preparation.

